# Supplementary material for: Netcombin: An algorithm for constructing optimal phylogenetic network from rooted triplets
Source: PLoS One. 2020 Sep 18;15(9):e0227842. doi: 10.1371/journal.pone.0227842 (PMC7500971; doi:10.1371/journal.pone.0227842)
Supplement: S4 File — (ZIP) [file pone.0227842.s004.zip › SequenceData-Triplet/sequenceData-10-20-30-0-Triplet/length 1000, numseq 40, rate 10^-7/4 seq.pdf]

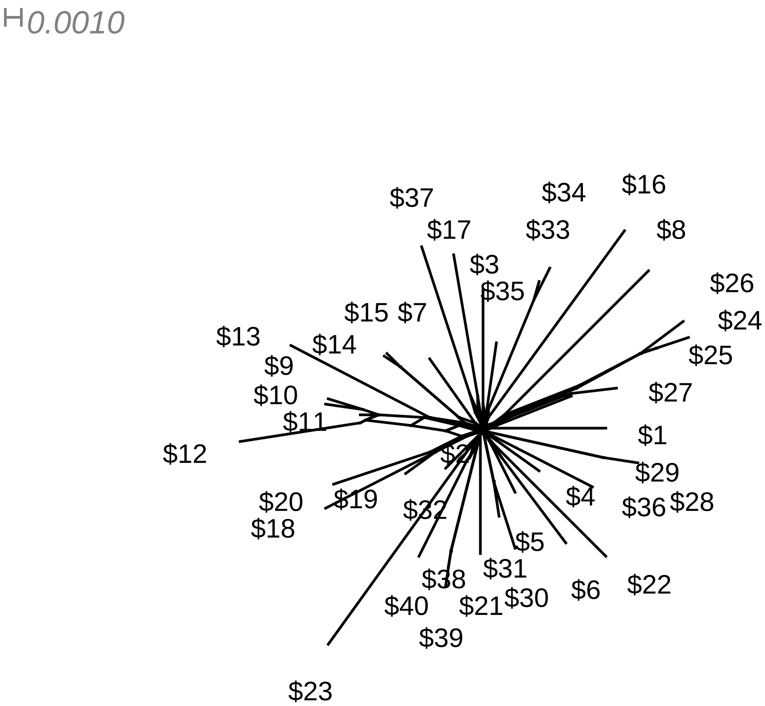
0.0010
$37
$34
$16
$17
$33
$8
$3
$35
$26
$15
$7
$24
$13
$14
$25
$9
$27
$10
$11
$1
$12
$2
$29
$20
$19
$4
$36
$28
$32
$18
$5
$31
$38
$30
$6
$22
$40
$21
$39
$23
